# Septic Shock and Multiorgan Failure Related to Liver Abscess Caused by Fish Bone Penetration of Duodenal Wall

**DOI:** 10.1155/crgm/8462610

**Published:** 2025-11-12

**Authors:** Md. Atiquzzaman, Injamam Ull Haque, Md Motiul Islam, Tarikul Hamid, Mohammad Rabiul Halim, Kazi Nuruddin Ahmed, Sadia Jesmin, Rajib Hasan, Quazi Abdullah Al Masum, Md. Ashikuzzaman Sohan

**Affiliations:** ^1^Department of Critical Care Medicine, Asgar Ali Hospital, Dhaka, Bangladesh; ^2^Department of Critical Care Medicine, Central Police Hospital, Dhaka, Bangladesh; ^3^Department of Gastroenterology and Hepatology, Asgar Ali Hospital, Dhaka, Bangladesh

**Keywords:** fish bone, foreign body, liver abscess, multiorgan failure, pyogenic, septic shock

## Abstract

We report an intriguing case of an 80-year-old male presented with high-grade fever and severe upper abdominal pain. An abdominal ultrasound indicated a deep-seated abscess in the Segment IV of liver. An elusive diagnosis was made after Contrast Enhanced Computed Tomography (CECT) scan of whole abdomen which disclosed a sharp, pointed fish bone accommodating between the distal part of gastric antrum and cavitary lesion of liver. In the operative room, an upper GI endoscopy revealed a healed duodenal ulcer. An explorative laparotomy was performed subsequently, and the perihepatic abscess cavity was drained along with the removal of fish bone about 3.15 cm in length from perihilar region without any damage to the adjoining structures. His management was centered around IV antibiotics, imaging, and subsequent surgical drainage. The gentleman made a good recovery.

## 1. Introduction

Pyogenic liver abscess (PLA) infrequently leads to hepatobiliary sepsis and eventually septic shock and multiorgan failure. It accounts for 13% of all intra-abdominal abscesses [1]. Cholangitis, systemic sepsis complicated with hematogenous spread, and trauma are frequently levelled as the main culprits behind PLA [2]. Prognosis of those abscesses is determined by early diagnosis, multidisciplinary care, and timely intervention. However, successful recovery in elderly population with multiorgan failure could be troublesome due to high morbidity and mortality. Here, we describe a successful case of PLA in an 80-year-old male who presented with high-grade fever and abdominal pain for 5 days. Later on, he developed hypotension with subsequent septic shock and multiorgan failure.

## 2. Case Presentation

An 80-year-old man was evaluated at this hospital because of fever and abdominal pain. The gentleman had high-grade continued fever, highest recorded at 104°F with chills, and rigor. Fever subsided with antipyretic medications.

He also experienced upper abdominal pain which became more persistent and increased in severity with time. The pain was confined to the epigastric and right upper quadrant of the abdomen and progressively became more severe, prompting him to seek care in the emergency room (ER). In the ER, the patient described the pain as sharp and rated it at 9 on a scale of 0 to 10 (with 10 indicating the most severe pain).

On examination, the temporal temperature was 103°F, the heart rate was 108 beats per minute, the blood pressure was 108/78 mm Hg, the respiratory rate was 19 breaths per minute, and the oxygen saturation was 96% while the patient was breathing ambient air. He had dry mucous membranes. He had diffuse abdominal tenderness with guarding on palpation of the epigastrium and right upper quadrant. Bowel sounds were present on auscultation. The BMI was 22.95 kg/m^2^. The patient reported no chest pain, dyspnea, diarrhea, vomiting, dysuria, cough, and neck stiffness. No lymphadenopathy was noted.

A review of other systems was negative. He denied any alcohol, illicit drug, or tobacco use. The patient's medical history was notable for hypertension for 30 years, and he underwent Coronary Artery Bypass Grafting (CABG) 10 years back due to triple vessel disease. He had good compliance with his usual medications which included clopidogrel, rosuvastatin, and telmisartan.

The gentleman was admitted in in-patient department (IPD) under the department of gastroenterology. IV fluids and antibiotics (IV piperacillin-tazobactam and metronidazole) were started. However, 8 h after admission, he developed hypotension (70/50 mm Hg), tachycardia (118 b/min) along with raised temperature (102°F), and new onset altered mentation. His national early warning score (NEWS) 2 score was found to be 10 requiring escalation of care to intensive care unit (ICU).

**Figure 1:**
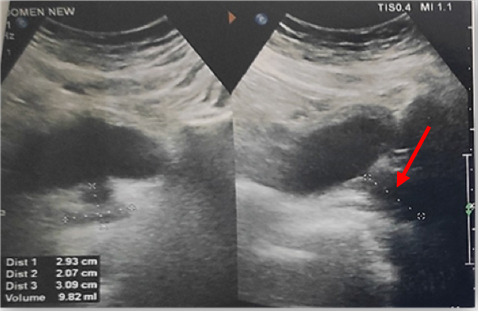
Liver ultrasound images showed a hypoechoic focal area with irregular outline (red arrow, an abscess cavity), but there was no demonstrable foreign body.

During ICU stay, initial resuscitative measures were undertaken. Point-of-care ultrasound (POCUS) showed a collapsed IVC, no pericardial effusion, and no B lines. USG of whole abdomen revealed a heterogeneous hypoechoic focal area of about 4.0 × 3.9 cm with irregular outline in the Segment IV of liver, corresponding an abscess cavity (Figure 1).

Initial blood reports showed the white cell count was 18,750/μL, procalcitonin level was 80.38 ng/mL, platelet count was 74,000/μL, c-reactive protein was 404.5 mg/dL, and serum creatinine level was 3.96 mg/dL with low hemoglobin. Other laboratory test results are shown in Table 1.

**Figure 2:**
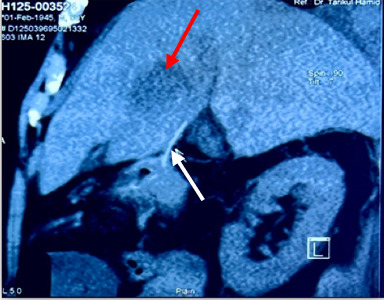
Abdominal CT-sagittal section showing a foreign body (white arrow) between gastric antrum and hepatic lesion (red arrow).

Later, a CECT scan of whole abdomen described an ill-defined irregular marginated cavitating multiloculated fluid containing lesion measuring about 4.18 × 4.47 × 3.58 cm in segment IV of liver anterior aspect of gallbladder abutting wall with faint enhancement of the wall, septations after contrast. It also revealed a linear radio-opaque foreign body measuring about 3.15 cm in length between distal gastric antrum and hepatic lesion (Figure 2).

A multidisciplinary team approach was undertaken. We took the dietary history again from the attendants and found that he might have a history of fish ingestion 7 days back and subsequently a fish bone might have accidently pierced the gut and penetrated the liver, causing local inflammatory reaction and forming abscess with clinical features. A decision was taken for upper GI endoscopy.

In the meantime, our patient developed acute kidney injury (AKI) requiring renal replacement therapy in the form of sustained low-efficiency dialysis (SLED), altered coagulation profile requiring multiple units of blood products, thrombocytopenia probably related to sepsis, Type 2 MI, and septic shock on vasopressors.

**Figure 3:**
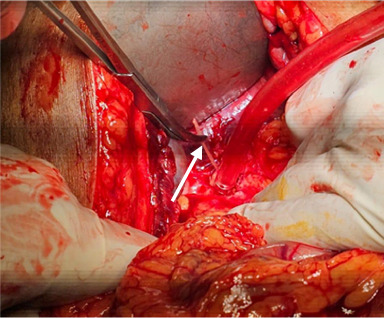
Removal of fish bone during laparotomy (white arrow).

After determining the patient's good tolerance to intervention, he was taken to the endoscopy suite. An endoscopy of upper GIT revealed a healed duodenal ulcer but no foreign body. Later on, explorative laparotomy was performed, and an abscess cavity was identified with formation of granulation tissue in the perihepatic region. Adequate drainage with curettage was done, and the fish bone was removed (Figure 3). He was successfully extubated the very next day. Our patient demonstrated significant clinical improvement with resolution of symptoms and normalization of blood test profile in the following couple of days. Septic shock resolved eventually.

The drainage tube was therefore removed on the 7th postoperative day. The patient was transferred under the department of hepatobiliary and gastrointestinal surgery in stable condition and later on discharged from hospital.

In this case reporting, we kept in mind ethical considerations like protection of privacy of the patient and took informed consent.

## 3. Discussion

PLA arises from certain pathogenic organisms such as bacteria, fungi, and parasites. Most common infectious routes are biliary, portal vein, hepatic artery, and direct penetrating injury to the liver [3]. However, PLA due to foreign body (fish bone) penetration of the GI tract is not a common scenario [4]. Overlooked or forgotten emigration of a foreign body from the GI tract is amongst the unusual causes of these abscesses. The most commonly found foreign bodies are fish bone (44%), toothpick (29%), and chicken bone (8%) [5].

Most foreign bodies about 80%–90% transit through the GI tract within 7 days without causing any harm if they pass through the lower esophageal sphincter [3, 6, 7]. Stomach and duodenum are the most common sites of penetration with most dislodgement occurring in the left lobe of liver [6, 8]. The very first case report of hepatic abscess following GI perforation by ingesting foreign body was reported by Lambert and colleagues in 1898, and since then, only just above 60 cases have been reported in the literature [6, 8].

Accidental ingestion of fish bones is most often encountered in Asian and Pacific populations, where fish is commonly consumed [9, 10]. The sharp and pointy ends of the fish bones pose a threat to GI perforation. Aged individuals struggle with food processing in the mouth related to impaired dentition, neurological problems, and sedative drugs [11].

For discovering a foreign body in GIT, CT scan is the preferred modality (60.2%) due to its top notch resolution and accuracy [6, 9, 12, 13]. Foreign bodies will appear as calcified linear radiopaque structures on CT [9]. Endoscopy could be done earlier to remove foreign bodies before its emigration, and healing of the mucosal layer has taken place [13, 14].

In our case, the patient developed septic shock with multiorgan failure. We promptly started IV antibiotics, and after achieving fitness for intervention, we went for exploratory laparotomy to salvage the foreign body and drainage of the abscess cavity [6, 10]. In the literature, laparotomy for salvaging fish bone was done in 37.04% cases [15].

PLA poses a great threat due to high morbidity and mortality. The mortality in a recent review of 17 cases of liver abscesses secondary to fish bone perforation was 17.6% [6]. The prominent cause of death remains septic shock [12].

However, it is obvious that early diagnosis and prompt treatment with multidisciplinary approach are important for PLA caused by foreign bodies complicated with septic shock and multiorgan failure.

## 4. Conclusion

Herein, we successfully treated an individual with PLA by a penetrating fish bone complicated with septic shock and multiorgan failure. A compatible dietary history is always inimitable to diagnostic accuracy. Imaging modalities (CT and/or USG) form the foundation stone for prompt definitive diagnosis. A multidisciplinary approach is a must for this type of complicated and rare cases to get the best possible therapeutic management.

## Figures and Tables

**Table 1:** Patient's laboratory tests results.

Variable	Reference range	On ICU admission	2 days after surgery
Hemoglobin (gm/dL)	13.0 to 17.0	10.1	8.5
White cell count (per μL)	4000 to 10,000	18,750	10,890
Platelet count (per μL)	150,000 to 410,000	74,000	98,000
Procalcitonin (ng/mL)	< 0.05	80.38	4.34
C-reactive protein (mg/L)	< 5	404.5	68.7
Serum urea (mg/dL)	15–45	139	62
Serum creatinine (mg/dL)	0.72–1.25	3.96	1.74
Serum Na^+^ (mmol/L)	137–145	131	148
Serum K^+^ (mmol/L)	3.5–5.1	3.62	3.90
Serum Cl^−^ (mmol/L)	98–107	97	112
Serum ALT (U/L)	< 55	82	45
Trop-I (ng/mL)	< 0.1	2.61	0.272
D-dimer (μg/mL)	< 0.5	100	13.2
Fibrinogen (mg/dL)	200–400	117.9	296.1
PT (sec), INR	10–16	19.1, 1.67	16.5, 1.4
aPTT (sec)	26–37	40.1	26
